# Personality and Post-traumatic Growth of Adolescents 42 Months after the Wenchuan Earthquake: A Mediated Model

**DOI:** 10.3389/fpsyg.2017.02152

**Published:** 2017-12-12

**Authors:** Yuanyuan An, Xu Ding, Fang Fu

**Affiliations:** ^1^School of Psychology, Nanjing Normal University, Nanjing, China; ^2^School of Social Development and Public Policy, Fudan University, Shanghai, China

**Keywords:** post-trauma growth, personality, coping style, social support, mediating effect

## Abstract

The aim of this study was to examine the relationship of teenagers’ post-traumatic growth (PTG) and personality and coping style by developing a mediating model with matched data from 772 adolescents. The sample consisted of 772 adolescents (mean age = 12.93, *SD* = 1.80) from several middle schools located in the areas that were most severely affected by the earthquake. Five factor model of personality, Coping Style Scale and Post-traumatic Growth Inventory were used to measure personality, coping and PTG of adolescents respectively. The results showed that the mean of PTG is 2.87 (*SD* = 0.93). Moreover, the relationship between personality and PTG is mediated by cognitive coping. The model’s fit indices indicated a good fit (CFI = 0.996, TLI = 0.962, NFI = 0.994, RMSEA = 0.055). Results showed that a positive cognition coping style mediated the relationship between personality and PTG.

## Introduction

In recent years, trauma research has paid as much attention to adolescent survivors as to adult survivors (e.g., Alisic et al., 2011; Hafstad et al., 2011; Kilmer et al., 2014). Post-traumatic growth (PTG), defined as a “positive change experienced as a result of the struggle with highly challenging life circumstances that represent significant challenges to the adaptive resources of the individual” (Tedeschi and Calhoun, 2004, p. 1), has received considerable attention in research literature on adolescence (Zoellner and Maercker, 2006; Clay et al., 2009; Taku, 2011). There is some debate about the PTG of adolescents. Although previous studies have laid a solid groundwork, several salient questions remain unanswered. First, is PTG defensive or self-presentational (McMillen, 2004). Some scholars insisted that respondents may attempt to paint a more positive picture of their lives. When people report growth in the immediate months following loss or trauma, some part of this growth might indicate the strategy of self-protection, which may be an important aspect of the growth process (Davis and McKearney, 2003). It is argued that growth takes time to emerge. Therefore, existence of PTG soon after the event may serve as a coping strategy in order to reduce distress, and more data collection points after a trauma is suggested to verify the nature of PTG (Hall et al., 2010; Kilmer et al., 2014). Moreover, the research interest has been exploring the impact mechanism of PTG following traumatic events (Kilmer et al., 2009; Kilmer and Gil-Rivas, 2010; Meyerson et al., 2011), in which the impact of personality and positive coping on PTG have been highlighted (Prati and Pietrantoni, 2009; Moore et al., 2015; Zerach, 2015).

Personality, a stable psychological resource with cross-situational stability and propensity, plays an important role in the development of PTG (Zerach, 2015). Costa and McCrae’s five factor model of personality (FFM; 1992) consists of extraversion, neuroticism, openness, agreeableness, and conscientiousness dimensions. Previous studies showed that all five dimensions of personality play important role in leading to negative outcomes or PTG following traumatic events (Meuser and Marwit, 2000; Linley and Joseph, 2004; Shakespeare-Finch et al., 2005; Moran et al., 2013; Moore et al., 2015). More specifically, neuroticism is often correlated to a pathogenic post-traumatic outcome (Affleck and Tennen, 1996; Watson and Hubbard, 1996), while extraversion is found to be positively associated with positive perceptions of trauma (Tedeschi and Calhoun, 1996); openness is associated with the tendency to use cognitive strategies, which in turn leads to higher scores in measures of PTG (Tedeschi and Calhoun, 1996; Edwards et al., 1998). Meanwhile, agreeable individuals are more likely to perceive positive changes after experiencing traumatic events, and conscientiousness is characterized by self-control and self-efficacy. Individuals with high conscientiousness tend to be more meticulous and self-disciplined, pursuing goals with high tenacity, which in turn facilitates PTG (Costa and McCrae, 1992; Tedeschi and Calhoun, 1996).

In addition to the examination of the relation between personality and PTG, efforts have been made to identify mechanisms through which individuals with certain personalities develop PTG or factors that influence this development (Zhou et al., 2015b). As personality traits are widely accepted as fundamental, it is of great interest to explore the proximal mechanisms through which personality influences PTG (Robinson and Marwit, 2006). Park and Folkman (1997) proposed a transactional model to explain the effecting mechanisms of PTG. In the transactional model, PTG is produced by the interaction of stable human traits and traumatic events; personality has a stable role in the prediction of PTG, and this effect is most likely to occur through the mediating effect of cognitive evaluation and response. Moran et al. (2013) proposed that the acquisition of PTG involves the use of adaptive coping skills in the face of trauma: in other words, adaptive coping skills such as positive appraisal facilitates PTG (Prati and Pietrantoni, 2009; Pietrzak et al., 2011).

Coping refers to the process individual dealing with the demands and emotions caused by stressful events (McCammon et al., 1988; Lazarus, 1999; Shakespeare-Finch et al., 2005). It is between stressors and individual health that appropriate coping can alleviate stress in individual situations of trauma. Personality-coping-outcome theory suggested that, personality influences coping in different manners in the context of stressful events, which further influences psychological outcomes (Zhou et al., 2017). The theory suggests that coping style mediates the relationship between personality and psychological outcomes. Some evidence showed that coping style mediates the relation between personality and PTG of adolescents. For instance, empirical research suggested that personality was significantly related to coping and personality may influence one’s coping style (Connor-Smith and Flachsbart, 2007). Moreover, other studies have shown that positive reappraisal of the traumatic event is not only critical to successful adaptation, but also a prerequisite for personal growth (Janoff-Bulman, 1992; Calhoun and Tedeschi, 1998; Prati and Pietrantoni, 2009). Therefore, the coping process is considered a mediating variable between individuals and post-trauma outcome (Calhoun et al., 2000).

### The Current Study

The current study focuses on adolescents that survived the Wenchuan earthquake. The Wenchuan earthquake was a destructive and violent earthquake that occurred in Sichuan, China. It was measured 8.0 on the Richter scale, and caused huge loss and casualties (Liu et al., 2011). Adolescents who face drastic developmental and psychosocial changes are considered to be particularly vulnerable when facing the extra changes induced by disasters parallel to this storm and stress period. Adolescents face emotional, cognitive, and behavioral challenges including managing their negative emotions, reflecting on existential questions raised by a disaster, and acting on new environmental demands (Foa et al., 2001; Milam et al., 2004). Therefore, it is essential to pay more attention to this population and figure out the long-term impact of earthquake experiences on adolescents.

Most studies have recruited relatively small convenience samples with similar traumatic exposure (Pietrzak et al., 2010; Tsai et al., 2015). Larger samples from different cultures is needed to further test the research assumptions of trauma field. An additional limitation of extant research on PTG is that recent studies examined the direct effects between personality, positive coping, and PTG; nevertheless, it is possible that personality impacts PTG through positive coping (Shakespeare-Finch et al., 2005). Therefore, it is important to go further to better understand the mechanisms of the PTG process and its implications for children and youth (Kilmer and Gil-Rivas, 2010; Kilmer et al., 2014).

We had two aims in this study: (1) to evaluate the prevalence of PTG in adolescents 42 months after the Wenchuan earthquake; and (2) to test the mediating role of positive cognitive coping between personality and PTG. We hypothesized that five dimensions of personality and positive coping will be significantly associated with PTG. Moreover, Tedeschi et al. (1998) proposed that personality associated with PTG indirectly through coping resources, and in this study, we further posited that positive cognition coping would mediate the relationship between five dimensions of personality and PTG.

## Materials and Methods

### Participants and Procedures

Data were collected as part of a large longitudinal research project of child survivors of the Wenchuan earthquake. 772 adolescents were recruited from several middle schools with severe earthquake exposure. Data in this study were collected 42 months after the earthquake. The mean age of the adolescents in this study was 12.93 years (*SD* = 1.80) and 414 (53.6%) were female and 358 (46.4%) were male.

The study was approved for ethical treatment of human subjects by the School of Psychology at Nanjing Normal University. The researchers carried out the survey without the presence of local teachers. The aim of the study and the rights of the participants were declared, and written consent forms were signed by the participants prior to the survey. As most of the participants was living in boarding school, which were often far from their homes, it is difficult for us to get written informed consent from their parents. Nevertheless, we asked them to call their parents for permission to take part in the survey. Participants started to fill in the questionnaires after they had oral permission from their parents. The assessment was performed by trained university students majored in psychology.

### Measures

#### Post-traumatic Growth Inventory

Post-traumatic growth was measured by Chinese version of the Post-traumatic Growth Inventory (PTGI; Zang, 2010). The original PTGI developed by Tedeschi and Calhoun (1996) consisted of five dimensions: personal strength, new possibilities, relating to others, appreciation of life, and spiritual change. The scale included 21 items, and the responses were made on a six-point scale ranging from 0 = *no change* to 5 = *very great degree of change*. As for the Chinese version of PTGI, the original scale was revised in three ways. First, some items were re-phrased to be easily understood by adolescent survivors of the Wenchuan earthquake. Second, one item on the spiritual change subscale (“I have a strong religious faith”) was revised to “I now have a better understanding of the importance of religious beliefs and behaviors,” as the majority of students in China identify as having no religious beliefs. Third, because the spiritual change subscale had only two items, an additional item (“I now have better appreciation of the power of supernatural beings”) was added to improve the reliability of the subscale. The Chinese version of scale demonstrated good internal consistency (alpha coefficient was 0.93) in the current sample.

#### Coping Style Scale

The Coping Style Scale was developed by Xiao and Xu (1996) and adapted for real-world post-traumatic situations experienced by Chinese middle school students. There are four main measurements in this scale as follows: positive cognition, avoidant coping, social support seeking, and abreaction coping. In the current survey, we used only the items that measured positive cognition coping. For each item, the respondents indicated their use (or lack of use) of a given coping strategy. The scale showed good internal consistency in this study (alpha coefficient was 0.79).

#### Personality Scale

The Personality Scale was measured by FFM and adapted for use in Chinese cultures (Zhou et al., 2000). The FFM includes five dimensions:extraversion, openness, agreeableness, conscientiousness, and neuroticism (Costa and McCrae, 1992). The responses were made on a five-point Likert-type scale. The internal consistency of FFM in this study was 0.908.

### Analysis Procedure

The statistical analysis was handled using SPSS 22.0, and included means, standard deviations (SD), and correlations of the four scales in the current study. Only 0.72% of the data was missing. Little’s Missing Completely at Random (MCAR) test suggested that the rate of missing data was equivalent across all measures (*p* > 0.05). Finally, AMOS 21.0 was adopted to test the hypothesized model and the mediation effects.

We also applied Harman’s single-factor test to examine common method bias (Podsakoff et al., 2003). All items relevant to the study were subjected to an exploratory factor analysis, and the un-rotated factor solution was examined to determine the number of factors that are necessary to account for the overall variance. This procedure these factors, while no single factor accounted for the majority of the covariance among the variables. Therefore, no significant common method bias existed in the current study.

## Results

As showed in **Table 1**, the mean of PTG is 2.87 (*SD* = 0.93). Next, the Pearson correlation among different variables are analyzed, and it is shown that personality, positive cognition coping, and PTG are significantly correlated with each other.

**Table 1:** Descriptive statistics and Pearson correlations between the variables.

Variables	*M*	*SD*	2	3	4	5	6	7
(1) Extraversion	2.34	0.80	0.58^∗∗^	0.50^∗∗^	0.35^∗∗^	-0.081^∗^	0.32^∗∗^	0.32^∗∗^
(2) Openness	2.30	0.74		0.56^∗∗^	0.53^∗∗^	0.051	0.38^∗∗^	0.28^∗∗^
(3) Agreeableness	2.66	0.67			0.68^∗∗^	-0.025	0.39^∗∗^	0.32^∗∗^
(4) Conscientiousness	2.12	0.71				-0.058	0.34^∗∗^	0.33^∗∗^
(5) Neuroticism	1.96	0.81					0.041	-0.058
(6) Positive cognition coping	2.48	0.84						0.32^∗∗^
(7) PTG	2.87	0.93						

^∗∗∗^*p < 0.001; ^∗∗^*p* < 0.01; ^∗^*p* < 0.05*.

**FIGURE 1:**
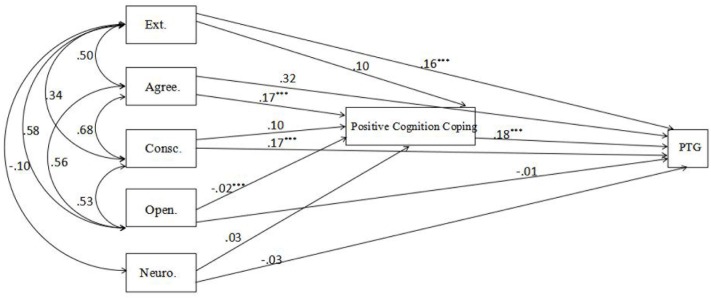
Mediation model of the relationships between the Big Five, Social Support, Positive Cognition Coping, and Post-traumatic Growth Inventory. Ext., extraversion; Agree., agreeableness; Consc., conscientiousness; Open., openness; Neuro., neuroticism. ^∗∗∗^*p* < 0.001.

A mediated model of the Big Five, positive cognition coping, and PTG was examined. The Big Five were taken as exogenous variables, and PTG and positive cognition coping as endogenous variables. We hypothesized that positive cognition coping would mediated the relationship between the Big Five and PTG. SEM was performed to examine the hypothesis by AMOS (Arbuckle and Wothke, 1999). Goodness of fit indices such as CFI, TLI, NFI, RMSEA were chosen to assess the model’s fit (Bentler, 1990). A good fit was indicated by the model fit indices: χ^2^/df = 3.295, Comparative Fit Index (CFI) = 0.996, Tucker-Lewis Index (TLI) = 0.962, Non-normed Fit Index (NFI) = 0.994, Root Mean Square Error of Approximation (RMSEA) = 0.055. The criteria for every indices was: CFI > 0.90, TLI > 0.90, NFI > 0.90, RMSEA > 0.08. The model, shown in **Figure 1**, was therefore accepted.

A significant indirect effect was necessary for mediation assumption (Preacher and Hayes, 2004). A mediational test was conducted to reveal the indirect effects so that the mediating role of positive cognition coping between personality and PTG can be determined. **Table 2** shows different mediated pathway effects in this model.

**Table 2:** Indirect and direct effects of the Big Five, Positive Cognition Coping on Post-traumatic Inventory Growth (*N* = 772).

Independent	Mediator	Dependent	Mediation		Mediation	Direct effect of IV
variable		variable	confidence interval		*p*-value	on DV *p*-value
			Lower	Upper		
Extraversion	PCC	PTG	0.012	0.071	^∗^	^∗∗∗^
Openness	PCC	PTG	0.004	0.052	^∗∗∗^	0.851
Agreeableness	PCC	PTG	0.004	0.064	^∗∗∗^	0.545
Conscientiousness	PCC	PTG	0.008	0.063	^∗^	^∗∗∗^
Neuroticism	PCC	PTG	0.083	0.218	0.137	0.682

*PCC, Positive Cognition Coping; PTGI, Post-traumatic Growth Inventory*.

*^∗∗∗^*p* < 0.001; ^∗∗^*p* < 0.01; ^∗^*p* < 0.05*.

In regard to PTG, test results suggested that higher extraversion predicted greater PTG partially through increased positive cognition coping (with the indirect effect = 0.040, 95% CI [0.012, 0.071]). Conscientiousness also indirectly predicted greater PTG partially through increased positive cognition coping (with the indirect effect = 0.032, 95% CI [0.008, 0.063]). Agreeableness indirectly predicted greater PTG fully through increased positive cognition coping (0.171, 95% CI [0.004, 0.064]). Additionally, openness indirectly predicted greater PTG fully through increased positive cognition coping (0.167, 95% CI [0.004, 0.052]).

In conclusion, these results indicated that positive cognition coping fully mediated the effects of agreeableness and openness on PTG. Besides, the effects of extraversion and conscientiousness on PTG were direct but also indirect, partially mediated by positive cognition coping.

## Discussion

The goal of this study was to examine the level of PTG 42 months after the earthquake as well as to test a comprehensive model including positive cognition coping as mediating variables in the relationship between personality and PTG among adolescent survivors.

The study found that 42 months after the Wenchuan earthquake, adolescents had a medium level of PTG. Previous research found that adolescents had a higher level of PTG in the short term. For instance, Jia et al. (2015) presented in their research that 12 months after the Wenchuan earthquake, the mean score of PTG for adolescents was 3.10 (*SD* = 0.91). As time went by, the level of PTG went down a little bit; as it is shown in our study, the mean score was 2.87 (*SD* = 0.93). Meanwhile, Zhou et al. (2015a) also examined the level of PTG for adolescent survivors 5.5 years after the Wenchuan earthquake and found the level of PTG was 2.74 (*SD* = 0.91). The results of this study may provide some clues to the question of whether PTG is a self-defense coping strategy or a real positive change. One year after the earthquake, the level of PTG was higher. This implies that in the early stages of adaptation, seeking growth may serve as a defense to cope with distress. In other words, soon after the earthquake, PTG could have served as both a coping strategy and real growth. However, the level of PTG went down as time went by; nevertheless, adolescents may experience true growth eventually. It implies that at the very beginning, PTG may mainly serve as a self-enhancing illusion in order to help individuals reduce emotional distress; however, the real growth shall come about if they stick to this “positive illusion” long enough.

In addition, the SEM showed that positive cognition coping fully mediated the effects of agreeableness and openness on PTG. Besides, the effects of extraversion and conscientiousness on PTG were direct but also indirect, partially mediated by positive cognition coping. That is, the impact of extraversion and conscientiousness on PTG worked not only in a direct way, but improved PTG with positive cognition coping.

The results verify the interaction model; namely, positive changes after experiencing trauma are produced by the interaction of stable human traits and a traumatic event. Personality on the forecast of PTG has a stable effect, and this effect is likely to work through the intermediary role of cognitive appraisal and coping. Coping is cognitive and behavioral patterns of individuals when faced with stressful situations and crises. Coping not only can ease the emotional trauma caused by the crisis, but also can promote youth to have deeper cognitive processing and active mediation, and take on well-adapted coping strategies so that they will have a higher potential to develop and a higher level of physical and mental growth. Positive coping has a high positive correlation with multiple dimensions of PTG and could significantly predict growth (Bellizzi and Blank, 2006).

### Mechanisms of Coping on PTG

McCrae and Costa (1986) proposed that personality differences may relate to differences in responses to exposure to traumatic events. The results of this study further supported this assumption. Specifically speaking, positive cognition fully mediated the relationship between agreeableness, openness, and PTG. Adolescents who scored higher on agreeableness was more altruistic and considerate, while adolescents with open personalities were more creative and curious. It seems that both characteristics had no direct effect on PTG; nevertheless, individuals with agreeable personality are related to interpersonal competency and they may learn positive coping from other persons. With regard to openness, individuals with an open personality tend to be more optimistic and employ positive coping to deal with difficulties, which in turn predict higher levels of PTG.

Previous studies reported that individuals with an extroverted personality are more capable of creating a supportive environment and making use of different social resources to cope with difficulties, which can help them find benefits in difficult situations (Jia et al., 2015). The core of consciousness is strict self-discipline, persistence, and impulse control, which have ongoing protection effects and reduce the risk of individual psychological problems while prompting individuals’ positive growth and well-being (Schnurr et al., 1993; Schwarzer et al., 2006). Meanwhile, conscientiousness is closely linked to positive cognition. Individuals with high conscientiousness tend to have a strict self-discipline and strong willpower, so in trauma context, they prefer to overcome the negative results caused by traumatic events and generate positive cognitive evaluations (Shakespeare-Finch et al., 2005). This urges teens to engage in deep cognitive processing and active rumination on the event in order to find hidden meaning behind it. Therefore, it is helpful to reconstruct the meaning of the world and develop PTG if the individual with a conscientious personality is inclined to adopt a positive cognitive coping style.

Although this study systematically explores the relationship between personality and PTG and the effect of coping on this relationship, there are still some limitations. First, as a cross-sectional study, it fails to examine the development of teenagers’ PTG at different times, characteristics of the change of PTG over time, and so on. Second, some relatively specific variables in the field of PTG, such as mediation and meaning seeking, were not explored in this study.

In future studies, researchers can use a track design to explore the development characteristics of teenagers’ PTG after a natural disaster, taking cultural background factors and characteristics of adolescent psychological development into account, while also focusing on the relationship between PTG and other psychological health variables, and exploring psychological rehabilitation and interventions in a positive perspective.

## Author Contributions

YA designed the current study. XD collected and analyzed data. FF wrote this article.

## Conflict of Interest Statement

The authors declare that the research was conducted in the absence of any commercial or financial relationships that could be construed as a potential conflict of interest.

## References

[B1] Arbuckle J. L. Wothke W. (1999). *Amos 4.0 User’s Guide*, 6th Edn Chicago, IL: SmallWaters Corporation.

[B2] Affleck G. Tennen H. (1996). Construing benefits from adversity: adaptotional significance and dispositional underpinnings. *J. Pers.* 64 899–922. 10.1111/j.1467-6494.1996.tb00948.x 8956517

[B3] Alisic E. Jongmans M. J. van Wesel F. Kleber R. J. (2011). Building child trauma theory from longitudinal studies: a meta-analysis. *Clin. Psychol. Rev.* 31 736–747. 10.1016/j.cpr.2011.03.001 21501581

[B4] Bellizzi K. M. Blank T. O. (2006). Predicting posttraumatic growth in breast cancer survivors. *Health Psychol.* 25 47–56. 10.1037/0278-6133.25.1.47 16448297

[B5] Bentler P. M. (1990). Comparative fit indexes in structural models. *Psychol. Bull.* 107 238–246. 10.1037//0033-2909.107.2.238 2320703

[B6] Clay R. Knibbs J. Joseph S. (2009). Measurement of posttraumatic growth in young people: a review. *Clin. Child Psychol. Psychiatry* 14 411–422. 10.1177/1359104509104049 19515756

[B7] Calhoun L. G. Tedeschi R. G. (1998). “Posttraumatic growth: future directions,” in *Posttraumatic Growth: Positive Changes in the Aftermath of Crisis*, eds Tedeschi R. G. Park C. L. Calhoun L. G. (Mahwah, NJ: Erlbaum), 215–238. 10.4324/9781410603401

[B8] Calhoun L. G. Cann A. Tedeschi R. G. McMillian J. (2000). A correlational test of the relationship between post-traumatic growth, religion, and cognitive processing. *J. Trauma. Stress* 13 521–527. 10.1023/a:1007745627077 10948491

[B9] Connor-Smith J. K. Flachsbart C. (2007). Relations between personality and coping: a meta-analysis. *J. Pers. Soc. Psychol.* 93 1080–1107. 10.1037/0022-3514.93.6.1080 18072856

[B10] Costa P. T. McCrae R. R. (1992). Four ways five factors are basic. *Pers. Individ. Differ.* 13 653–665. 10.1016/0191-8869(92)90236-I

[B11] Davis C. G. McKearney J. M. (2003). How do people grow from their experience with trauma or loss? *J. Soc. Clin. Psychol.* 22 477–492. 10.1521/jscp.22.5.477.22928

[B12] Edwards J. A. Weary G. Reich D. A. (1998). Casual uncertainty: factor structure and relation to the Big Five personality factors. *Pers. Soc. Psychol. Bull.* 24 451–472. 10.1177/0146167298245001

[B13] Foa E. B. Johnson K. M. Feeny N. C. Treadwell K. R. (2001). The child PTSD symptom scale: a preliminary examination of its psychometric properties. *J. Clin. Child Psychol.* 30 376–384. 10.1207/S15374424JCCP3003-9 11501254

[B14] Hafstad G. S. Kilmer R. P. Gil-Rivas V. (2011). Posttraumatic growth among norwegian children and adolescents exposed to the 2004 tsunami. *Psychol. Trauma. Theory Res. Pract. Policy* 3 130–138. 10.1037/a0023236 20553518

[B15] Hall B. J. Hobfoll S. E. Canetti D. Johnson R. J. Palmieri P. A. Galea S. (2010). Exploring the association between posttraumatic growth and PTSD: a national study of Jews and Arabs following the 2006 Israeli-Hezbollah war. *J. Nerv. Ment. Dis.* 198 180–186. 10.1097/nmd.0b013e3181d1411b 20215994 PMC3652393

[B16] Janoff-Bulman R. (1992). *Shattered Assumptions.* New York, NY: The Free Press.

[B17] Jia X. Ying L. Zhou X. Wu X. Lin C. (2015). Effects of extraversion, social support and post-traumatic stress disorder on post-traumatic growth among adolescent survivors of the Wenchuan earthquake. *PLOS ONE* 10:e0121480. 10.1371/journal.pone.0121480 25815720 PMC4376870

[B18] Kilmer P. R. Gil-Rivas V. (2010). Exploring post-traumatic growth in children impacted by hurricane katrina: correlates of the phenomenon and developmental considerations. *Child Dev.* 81 1211–1227. 10.1111/j.1467-8624.2010.01463.x 20636691 PMC2907541

[B19] Kilmer R. P. Gil-Rivas V. Griese B. Hardy S. J. Hafstad G. S. Alisic E. (2014). Posttraumatic growth in children and youth: clinical implications of an emerging research literature. *Am. J. Orthopsychiatry* 84 506–518. 10.1037/ort0000016 25110973

[B20] Kilmer R. P. Gil-Rivas V. Tedeschi R. G. Cann A. Calhoun L. G. Buchanan T. (2009). Use of the revised posttraumatic growth inventory for children. *J. Trauma. Stress* 22 248–253. 10.1037/t22273-000 19462437 PMC2827205

[B21] Linley P. A. Joseph S. (2004). Positive change following trauma and adversity: a review. *J. Trauma. Stress* 17 11–21. 10.1023/b:jots.0000014671.27856.7e 15027788

[B22] Lazarus R. S. (1999). Hope: an emotion and a vital coping resource against despair. *Soc. Res.* 66 653–678.

[B23] Liu M. Wang L. Shi Z. Zhang Z. Zhang K. Shen J. (2011). Mental health problems among children one-year after sichuan earthquake in China: a follow-up study. *PLOS ONE* 6:e14706. 10.137/journal.pone.0014706 21373188 PMC3044135

[B24] McMillen J. C. (2004). Posttraumatic growth: What’s it all about? *Psychol. Inq.* 15 48–52.

[B25] Meuser M. T. Marwit J. S. (2000). An integrative model of personality, coping, and appraisal for the prediction of grief involvement in adults. *OMEGA* 40 375–393. 10.2190/p6bm-qu6c-6xy9-bnum

[B26] Meyerson D. A. Grant K. E. Smith Carter J. Kilmer R. P. (2011). Posttraumatic growth among children and adolescents: a systematic review. *Clin. Psychol. Rev.* 31 949–964. 10.1016/j.cpr.2011.06.003 21718663

[B27] Moore M. M. Cerel J. Jobes A. D. (2015). Fruits of trauma?post-traumatic growth among suicide-bereaved parents. *Crisis* 36 241–248. 10.1027/0227-5910/a000318. 26440620

[B28] Moran S. Schmidt J. Burker J. E. (2013). Post-traumatic growth and post-traumatic stress disorder in veterans. *J. Rehabil.* 79 24–43.

[B29] McCammon S. Durham T. W. Jackson Allison E. Williamson J. E. (1988). Emergency workers’ cognitive appraisal and coping with traumatic events. *J. Trauma. Stress* 1 353–372. 10.1007/bf00974770

[B30] McCrae R. R. Costa P. T. Jr. (1986). Personality, coping, and coping effectiveness in an adult sample. *J. Personal.* 54 385–404. 10.1111/j.1467-6494.1986.tb00401.x

[B31] Milam J. E. Ritt-Olson A. Unger J. B. (2004). Posttraumatic growth among adolescents. *J. Adolesc. Res.* 19 192–204. 10.1177/0743558403258273

[B32] Pietrzak R. H. Goldstein R. B. Southwick S. M. Grant B. F. (2011). Prevalence and axis I comorbidity of full and partial posttraumatic stress disorder in the united states: results from wave 2 of the national epidemiologic survey on alcohol and related conditions. *J. Anxiety Disord.* 25 456–465. 10.1097/jgp.0b013e31820d92e7 21168991 PMC3051041

[B33] Pietrzak R. H. Johnson D. C. Goldstein M. B. Malley J. C. Rivers A. J. Morgan C. A. (2010). Psychosocial buffers of traumatic stress, depressive symptoms, and psychosocial difficulties in veterans of Operations Enduring Freedom and Iraqi Freedom: the role of resilience, unit support, and postdeployment social support. *J. Affect. Disord.* 120 188–192. 10.1016/j.jad.2009.04.015 19443043

[B34] Podsakoff P. M. MacKenzie S. B. Lee J. Y. Podsakoff N. P. (2003). Common method biases in behavioral research: a critical review of the literature and recommended remedies. *J. Appl. Psychol.* 88 879–903. 10.1037/0021-9010.88.5.879 14516251

[B35] Prati G. Pietrantoni L. (2009). Optimism, social support, and coping strategies as factors contributing to posttraumatic growth: a meta-analysis. *J. Loss Trauma.* 14 364–388. 10.1080/15325020902724271

[B36] Park C. L. Folkman S. (1997). Stability and change in psychosocial resources during caregiving and bereavement in partners of men with aids. *J. Personal. Issues* 65 421–447. 10.1111/j.1467-6494.1997.tb00960.x 9226944

[B37] Preacher K. J. Hayes A. F. (2004). SPSS and SAS procedures for estimating indirect effects in simple mediation models. *Behav. Res. Method* 36 717–731. 10.3758/bf03206553 15641418

[B38] Robinson T. Marwit J. S. (2006). An investigation of the relationship of personality, coping, and grief intensity among bereaved mothers. *Death Stud.* 30 677–696. 10.1080/07481180600776093 16869060

[B39] Schnurr P. P. Rosenberg S. D. Friedman M. J. (1993). Change in MMPI scores from college to adulthood as a function of military service. *J. Abnorm. Psychol.* 102 288–296. 10.1037/0021-843x.102.2.288 8315141

[B40] Schwarzer R. Luszczynska A. Boehmer S. Taubert S. Knoll N. (2006). Changes in finding benefit after cancer surgery and the prediction of well-being one year later. *Soc. Sci. Med.* 63 1614–1624. 10.1016/j.socscimed.2006.04.004 16765495

[B41] Shakespeare-Finch J. Gow K. Smith S. (2005). Personality, coping, and post-traumatic growth in emergency ambulance personnel. *Traumatology* 11 325–334. 10.1528/trau.2005.11.4.325

[B42] Tedeschi R. G. Calhoun L. G. (2004). Post-traumatic growth: conceptual foundations and empirical evidence. *Psychol. Inq.* 15 1–18. 10.1207/s15327965pli1501-02

[B43] Taku K. (2011). Commonly-defined and individually-defined posttraumatic growth in the US and Japan. *Pers. Soc. Psychol.* 51 188–193. 10.1016/j.paid.2011.04.002

[B44] Tedeschi R. G. Calhoun L. G. (1996). The post-traumatic growth inventory: measuring the positive legacy of trauma. *J. Trauma. Stress* 9 455–471. 10.1007/bf02103658 8827649

[B45] Tsai J. El-Gabalawy R. Sledge W. H. Southwick S. M. Pietrzak R. H. (2015). Post-traumatic growth among veterans in the USA: results from the national health and resilience in veterans study. *Psychol. Med.* 45 165–179. 10.1017/s0033291714001202 25065450

[B46] Tedeschi R. G. Tedeschi R. G. Park C. L. Calhoun L. G. (1998). *Posttraumatic Growth: Positive Changes in the Aftermath of Crisis.* Abingdon: Routledge 10.4324/9781410603401

[B47] Watson D. Hubbard B. (1996). Adaptational style and dispositional structure: coping in the context of the five-factor model. *J. Pers.* 64 737–774. 10.1111/j.1467-6494.1996.tb00943.x

[B48] Xiao J. Xu X. (1996). Study on the validity and reliability of “COPE.” *Chin. Ment. Health J.* 10 164–168.

[B49] Zoellner T. Maercker A. (2006). Posttraumatic growth in clinical psychology—A critical review and introduction of a two component model. *Clin. Psychol. Rev.* 26 626–653. 10.1016/j.cpr.2006.01.008 16515831

[B50] Zerach G. (2015). Secondary growth among former prisoners of war’s adult children: the result of exposure to stress, secondary traumatization, or personality traits?. *Psychol. Trauma. Theory Res. Pract. Policy* 7 313–323. 10.1037/tra0000009 26147516

[B51] Zang W. W. (2010). *The Comparative Study of Post-Traumatic Growth and Post-Traumatic Stress Disorder of Adolescents that Survived in the Wenchuan Earthquake.* Ph.D. dissertation, Peking University, Beijing.

[B52] Zhou Y. Y. Li D. P. Li X. Wang Y. H. Zhao L. Y. (2017). Big five personality and adolescent internet addiction: the mediating role of coping style. *Addict. Behav.* 64 42–48. 10.1016/j.addbeh2016.08.009 27543833

[B53] Zhou H. Niu L. Zou H. (2000). A development study on five-factor personality questionnaire for middle school students. *Psychol. Dev. Educ.* 16 48–54.

[B54] Zhou X. Wu X. Chen J. (2015a). Longitudinal linkages between post-traumatic stress disorder and post-traumatic growth in adolescent survivors following the Wenchuan earthquake in China: a three-wave, cross-lagged study. *Psychiatry Res.* 228 107–111. 10.1016/j.psychres.2015.04.024 25959264

[B55] Zhou X. Wu X. Fu F. An Y. (2015b). Core belief challenge and rumination as predictors of PTSD and PTG among adolescent survivors of the Wenchuan earthquake. *Psychol. Trauma. Theory Res. Pract. Policy* 7 391–397 10.1037/tra0000031 25793513

